# Development, Characterization, and Cellular Toxicity Evaluation of Solid Dispersion-Loaded Hydrogel Based on Indomethacin

**DOI:** 10.3390/polym16152174

**Published:** 2024-07-30

**Authors:** Zaid Dahma, Alexandra Ibáñez-Escribano, Cristina Fonseca-Berzal, Juan José García-Rodríguez, Covadonga Álvarez-Álvarez, Carlos Torrado-Salmerón, Santiago Torrado-Santiago, Paloma Marina de la Torre-Iglesias

**Affiliations:** 1 Department of Pharmaceutics and Food Technology, Faculty of Pharmacy, Complutense University of Madrid, Plaza Ramón y Cajal s/n, 28040 Madrid, Spain; zdahma@ucm.es (Z.D.); covadong@ucm.es (C.Á.-Á.); ctorrado@ucm.es (C.T.-S.); 2 Departamento de Microbiología y Parasitología, Faculty of Pharmacy, Complutense University of Madrid, Plaza Ramón y Cajal s/n, 28040 Madrid, Spain; alexandraibanez@ucm.es (A.I.-E.); crfonseca@ucm.es (C.F.-B.); jjgarc01@ucm.es (J.J.G.-R.); 3 Instituto Universitario de Farmacia Industrial (IUFI), Complutense University of Madrid, Plaza Ramón y Cajal s/n, 28040 Madrid, Spain

**Keywords:** indomethacin, low-substituted hydroxypropyl cellulose, hydroxypropyl guar, solid dispersion, hydrogels, cytotoxicity

## Abstract

Indomethacin (IND) as a non-selective cyclooxygenase 1 and 2 inhibitor administered orally causes numerous adverse effects, mostly related to the gastrointestinal tract. Moreover, when applied exogenously in topical preparations, there are obstacles to its permeation through the stratum corneum due to its low water solubility and susceptibility to photodegradation. In this work, solid dispersions (SDs) of IND with low-substituted hydroxypropyl cellulose (LHPC) were developed. The IND—SDs were incorporated into a hydroxypropyl guar (HPG) hydrogel to enhance drug solubility on the skin. The hydrogels were characterized by scanning electron microscopy (SEM), differential scanning calorimetry (DSC), powder X-ray diffraction (XRPD), Fourier-transform infrared spectroscopy (FTIR), viscosity, drug release, and unspecific cytotoxicity in mammalian cells. SEM showed a highly porous structure for SD hydrogels. DSC and XRPD studies showed that amorphous IND species were formed; therefore, these hydrogels exhibited superior drug release in comparison with IND raw material hydrogels. FTIR evidenced the presence of the hydrogen bond in the SD hydrogel. The rheology parameter viscosity increased across gels formulated with SDs in comparison with hydrogels with pure IND. In addition, IND—SD hydrogels combine the advantages of a suitable viscosity for dermal use and no potentially hazardous skin irritation. This study suggests that the formulated IND—SD hydrogels represent a suitable candidate for topical administration.

## 1. Introduction

Indomethacin (IND) is a nonsteroidal anti-inflammatory drug (NSAID) used to treat inflammation and pain in several autoimmune diseases [1,2,3]. Due to the frequent gastrointestinal issues associated with oral IND administration, alternative delivery methods are essential. Topical administration, for instance, offers several benefits over oral administration: it allows for the targeted delivery of the active ingredient to a specific site, bypasses the first-pass metabolism, reduces gastrointestinal side effects, and improves patient compliance [4]. For these reasons, the topical administration of IND or other topical NSAIDs is recommended for the treatment of inflammation, edema, and mild-to-moderate acute pain and for the relief of symptoms of arthritis (osteoarthritis and rheumatoid arthritis) or gout, such as inflammation, swelling, stiffness, and joint pain [5]. IND has low water solubility, so enhancing its solubility and dissolution is essential to improve its transport into the skin [4].

Different strategies such as the development of nanocrystals and the elaboration of solid dispersions improve the solubility and dissolution rate of IND [6,7]. In amorphous IND solid dispersions, the dissolution of this poorly water-soluble drug from SDs is a complicated process. Many factors, such as carrier type, drug physical state, granule particle size, and density, can affect the dissolution [8]. Different amorphous solid dispersions have been studied using different excipients like polyethylene glycol 4000 and Gelucire^®^ 50/13 [9], lactose [10], polyvinylpyrrolidone (PVP K25) [11], poloxamer 188 and PVP K30 [8], Eudragit^®^ EPO and hydroxypropyl methylcellulose (HPMC) [12], glucose [13], Gelucire^®^ 50/13 and Gelucire^®^ 48/16 [14], HPMC and Kollicoat^®^ IR [15], kaolin [16], and polyvinylpyrrolidone/vinyl acetate (Kollidon^®^ VA 64) [17]. Additionally, different parameters have been used to improve the solubility of poorly soluble drugs, incorporating the hydrophilic characteristics of the carrier [8], and the decrease in crystallinity, as seen by XRPD and DSC studies [8,9,11,13,14,15,16]. Previous studies have shown many amorphous solid dispersions utilizing low-substituted hydroxypropyl cellulose (LHPC) as a hydrophilic excipient. LHPC, known for its inert nature, functions as a disintegrant and improves the wettability and dispersibility of solid dispersions. These characteristics can potentially influence the dissolution behavior of the drug on the skin [18,19,20,21].

In developing hydrogel formulations, altering properties like water solubility, viscosity, and polymer chain length can influence how the drug is released [22]. Hydrogels composed of natural, semi-synthetic, or synthetic polymers such as chitosan, alginate, xanthan gum, hydroxypropyl methyl cellulose polymers, and partially hydrolyzed polyvinyl alcohol [22,23,24,25,26,27] have been used to obtain an immediate drug release. Amorphous SDs with different carriers, like different poloxamers, polysaccharides, polyvinylpyrrolidone (PVP), and an acrylic acid homopolymer (Carbopol 940), were employed to improve the dissolution of poorly soluble drugs from topical hydrogels [28,29,30,31,32]. A great number of topical hydrogel formulations perform their in vitro release investigations at pH values ranging from 5.0 to 7.4 [11,15,16,17]. In the present study, pH 5.8 was selected for the solubility and release rate analyses to enable comparison with various research on the topical administration of hydrogels [14,33]. Previous studies indicate that drugs like meloxicam exhibit strong hydrophobicity, which has been related to negative apparent dissolution entropies observed in neat water and could alter the surface energy, producing significant changes in surface wettability that contribute to the agglomeration process [33]. Other studies indicate that an improvement in surface wettability has been previously observed in solid dispersions with hydrophobic drugs, through the use of high percentages of surfactants [19]. In this work, we propose to improve the wettability of IND by adding low amounts of sodium dodecyl sulfate (SDS) as a humectant and LHPC as a hydrophilic polymer in different ratios to enhance the performance of hydrogels containing solid dispersions of indomethacin.

The objective of this study is to develop hydrogels containing IND solid dispersions that facilitate rapid topical drug release. The hypothesis is that utilizing LHPC as a hydrophilic carrier will enhance drug release rates, which will be investigated by examining various IND:LHPC ratios. Dissolution tests in a simulated skin medium (acetate buffer pH 5.8) will be conducted to evaluate the improvements in drug release from IND—SD hydrogels with different IND: LHPC proportions. Analytical techniques such as viscometry, scanning electron microscopy (SEM), Fourier-transform infrared spectroscopy (FTIR), X-ray powder diffraction (XRPD), and differential scanning calorimetry (DSC) will be employed to study viscosity properties, morphology, drug/polymer and polymer/polymer interactions, and the degree of crystallinity in the different IND solid dispersion (IND—SD) hydrogels. Additionally, the cytotoxicity of the IND—SD hydrogels will be assessed using four different mammalian cell lines. This study is significant as it aims to improve the efficacy and safety of topical drug delivery systems, potentially leading to better therapeutic outcomes.

## 2. Materials and Methods

### 2.1. Substances and Reagents

Indomethacin (IND) was purchased from Fagron Iberica SAU (Barcelona, Spain). Low-substituted hydroxypropyl cellulose (LHPC), with a hydroxypropoxy content of 5% to 16% and a molecular weight ranging from 30,000 to 150,000, was provided by Shin–Etsu^®^, Tokyo, Japan. Sodium dodecyl sulfate (SDS) was purchased from Fisher Scientific UK Ltd. (Leicestershire, UK). Hydroxypropyl guar (HPG), with a molecular weight of 90 KDa and a substitution level of 1.2, was obtained from Seppic^®^ (Barcelona, Spain). The water used in these experiments was obtained from a Milli–Q water purification system (Billerica, MA, USA). All other chemicals were at least of pharmaceutical grade.

### 2.2. Preparation of IND—SD Hydrogel Formulations

Different control hydrogels of HPG (H—HPG) were prepared by weighing 85 mg of the HPG polymer and dispersing it in 5 mL of pH 6.0 phosphate buffer, then stirring at 600 rpm until a homogeneous hydrogel blank was obtained.

IND solid dispersions (IND—SDs) were prepared via freeze-drying with LHPC as a carrier in various ratios (1:0, 1:1, 1:2.5, 1:5, 1:10, *w*/*w*). Each formulation used 20 mg of IND dissolved in 20 mL of an aqueous solution alkalized with 0.7 mL of 0.2 N NaOH. An amount of 0.4 mg of SDS was included as a humectant before adding the different amounts of LHPC. Then, samples were mixed at 2500 rpm for 2 min to obtain each solid dispersion. The different solid dispersion suspensions were frozen and freeze-dried. The formulations were then ground, sieved (0.125–0.500 mm), and stored with silica gel. A physical blend (PM—1:2.5) was also manually prepared using a ceramic vessel and a polymeric spatula. A blank formulation, with an IND:LHPC ratio of 0:2.5, was also formulated for different studies.

For the different hydrogels containing IND—RM, PM—1:2.5, or solid dispersions (SD—1:0, SD—1:1, SD—1:2.5, SD—1:5, and SD—1:10), 5 g of hydrogel blank was weighed. Then, an amount equivalent to 65 mg of IND for each solid dispersion was added and stirred at 600 rpm for 2 min. These hydrogels were used for viscosity and in vitro release rate studies. Finally, for differential scanning calorimetry (DSC), scanning electron microscopy (SEM), Fourier-transform infrared spectroscopy (FTIR), and cytotoxicity analyses, samples of different hydrogels with an HPG concentration of 1.75% (*w*/*v*)—including H—HPG, a hydrogel composed of pure IND (HIND—RM), physical mixture hydrogel (HPM—1:2.5), and hydrogels with IND—SDs at ratios of 1:0, 1:1, 1:2.5, 0:2.5, 1:5, and 1:10, underwent freeze-drying.

### 2.3. Scanning Electron Microscopy (SEM)

Samples were mounted on a double-faced adhesive tape and sputtered with a thin gold/palladium layer using a sputter coater Emitech K550X (Quorum Technologies; Lewes, UK). After coating, the hydrogel samples were analyzed with a Jeol JSM-6400 scanning electron microscope (Jeol Ltd., Peabody, MA, USA). All micrographs were the product of secondary electron imaging used for surface morphology identification at an accelerating voltage of 20 kV and a magnification of 500×.

### 2.4. Physicochemical Characterization

DSC scans for the different samples were obtained using an automated thermal analyzer system (QA-200 TA instrument, TA instruments, Elstree, UK). Temperature calibration was performed using Indium Calibration Reference Standard (transition point 156.60 °C). All dried samples were accurately weighed into aluminum pans and hermetically sealed with aluminum lids and heated from 25 to 320 °C at a rate of 10 °C/min under the constant purging of dry nitrogen at 100 mL/min. An empty pan was sealed and used as a reference with the same sample conditions.

The XRPD studies for the different formulations were performed on a Philips X’Pert-MPD X-ray diffractometer (Malvern Panalytical; Almelo, The Netherlands) at the CAI (Centro de Asistencia a la Investigación, Complutense University of Madrid, Spain). The samples were radiated using a monochromatized CuKα radiation (λ = 1.542 Å) and analyzed between the 5 and 40° (2θ) range at a step size of 0.04° and a time of 1 s per step.

The different samples for the Fourier-transform infrared spectroscopy (FTIR) study were prepared by weighing different amounts of the different formulations (equivalent to 2 mg of IND) and mixed with 200 mg of potassium bromide. The different samples were subjected to pressure at 10 T (Carver hydraulic press Model C-3912 (Wabash, IN, USA)). The analysis was conducted by using a Nicolet Nexus 670 FTIR spectrophotometer (Thermo Electron Corporation, Waltham, MA, USA). The spectra were obtained at a 2 cm^−1^ resolution with an average of 64 scans. The infrared region was analyzed in the range of 400–4000 cm^−1^.

### 2.5. Viscosity Study

The viscosities of hydrogel formulations containing 1.75% (*w*/*v*) HPG were evaluated using a Brookfield rheometer, model DV-III (Middleborough, MA, USA), equipped with a temperature-controlled probe and a 200 μm gap plate/plate configuration with a 40 mm diameter. Measurements were conducted at a constant temperature of 25 ± 0.5 °C. A viscosity assessment was performed to analyze the flow characteristics of several hydrogels: HPG blank hydrogel (H—HPG), IND raw material hydrogel (HIND—RM), physical mixture hydrogel (HPM—1:2.5), and solid dispersion hydrogel (HSD—1:2.5). Each sample underwent 55 measurements with a speed ramp from 0.5 to 700 s^−1^, each lasting 5 *s*. Each formulation underwent three replicates, and viscosity curves were graphed against shear rates to analyze their flow characteristics. The average viscosity (Pa·s) was calculated from the consistent shear section at 50 s^−1^ for each hydrogel.

### 2.6. Solubility Study

Solubility tests were performed on the following samples: IND raw material (IND—RM), physical mixture (PM—IND—1:2.5), and various solid dispersions (SD—1:0; SD—1:1; SD—1:2.5; SD—1:5; SD—1:10). Each sample, weighing 5 mg of IND or equivalent, was added to 5 mL of pH 5.8 buffer solution and agitated in a temperature-controlled bath at 32 ± 1 °C for 3 days. The solutions were then filtered, diluted with pH 5.8 buffer, and quantified at 267 nm using a UV–VIS JASCO^®^ V-730 spectrophotometer (Jasco International Co., Ltd.; Tokyo, Japan). Quantification was based on a calibration curve: y = 0.0588x (µg/mL) + 0.0109 (r^2^ = 0.9982), covering a concentration range of 1–15 µg/mL. Each measurement was triplicated, and error bars on graphs denote standard deviations.

### 2.7. In Vitro Drug Release

Drug release studies were carried out using hydrogels containing pure IND (HIND—RM), a physical mixture hydrogel (HPM—1:2.5), and hydrogels with solid dispersions (HSD—1:0; HSD—1:1; HSD—1:2.5; HSD—1:5; HSD—1:10). These investigations utilized the United States Pharmacopeia (USP) paddle over disk method (apparatus 5) with Erweka^®^ DT 80 dissolution equipment (Erweka GmbH; Langen, Germany), operating at 50 rpm and a temperature of 32.0 ± 0.5 °C in 300 mL of pH 5.8 phosphate buffer. Each disk contained 0.25 mL of various hydrogel formulations, equivalent to 3.2 mg of IND. Samples were filtered (Acrodisc^®^ HPVL 0.45 µm, Port Washington, NY, USA) at predetermined time points, and the cumulative release of IND was quantified at 267 nm using spectrophotometric analysis, following the method described in the solubility studies. Quadruplicate measurements were conducted at each time point, with error bars on graphs indicating standard deviations. Statistical comparisons were performed, with significance set at *p* < 0.05.

### 2.8. Cytotoxicity Analysis of Hydrogel Formulations

The unspecific cytotoxicity of the following samples IND raw material (IND—RM), hydrogels composed of pure IND (HIND—RM), physical mixture (HPM—1:2.5), solid dispersion (HSD—1:2.5), and HPG hydrogel (H—HPG) was evaluated in four different mammalian cells. These cells were purchased from the American Type Culture Collection (ATCC, Manassas, VA, USA).

Stock solutions of the compounds with 15% DMSO were prepared and added to the cultures to give a range of six different final concentrations in successive serial double dilutions (6.25–200 µm). The final concentration of DMSO solvent in the cell wells was less than 0.3% (*v*/*v*), which has no cytotoxic effect.

Epithelial cells from monkey (Vero CCL-81), murine macrophages J774, and human ovarian cells (HeLa) were grown in RPMI 1640 medium supplemented with 10% heat-inactivated fetal bovine serum (FBS) and antibiotics (penicillin 100 U/mL and streptomycin 100 μg/mL), [34,35], while murine fibroblasts L929 were maintained in MEM similarly supplemented [36]. All the cytotoxicity assays were performed following previously reported methods based on the fluorescent dye resazurin, with minor modifications [34,35,36]. Accordingly, Vero CCL81, J774 macrophages, and HeLa cells were seeded at 5 × 10^4^ cells/well (otherwise, L929 fibroblasts were seeded at 3 × 10^4^ cells/well) in 96-well flat-bottom microplates in a humidified atmosphere containing 5% CO_2_ at 37 °C. After cell attachment, cultures were exposed to various concentrations of the samples, ranging from 200 µM to 6.25 µM, and then incubated for another 24 h at the same conditions of temperature and humidity. Subsequently, 20 µL of 1 mM (2 mM for L929 cytotoxicity assays) resazurin stock solution prepared in PBS pH 7.0 was added to each well, and plates were incubated for 3 h at 37 °C. Finally, fluorescence intensity was measured in a Tecan infinite 200 multifunctional microplate reader (λexc 535 nm, λem 590 nm). All the compounds were tested in triplicate in three independent experiments (n = 3), and the results were expressed as the mean value of cytotoxicity ± standard deviation (SD). The controls of the compounds, medium, and cultures were included in all the plates.

### 2.9. Statistical Analysis

Differences between obtained values (mean ± SE) for the prepared formulae and the control formulae were calculated using a one-way analysis of variance (ANOVA), followed by Bonferroni post hoc tests in the case of a significant difference. A *p*-value less than 0.05 was considered the criterion for a statistically significant difference (Statgraphics^®^ Centurion, version 19).

## 3. Results and Discussion

### 3.1. Morphological Characterization of Hydrogels

The scanning electron microscopy (SEM) method was used to study the surface and morphological characteristics of different samples before and after their manufacturing process: IND—RM; PM—1:2.5; SD—1:1; SD—1:2.5; and the same solid dispersion without IND (SD—0:2.5) and the hydrogels; HIND—RM hydrogel; physical mixture HPM—1:2.5; and solid dispersion HSD—1:2.5 hydrogels.

**Figure 1:**
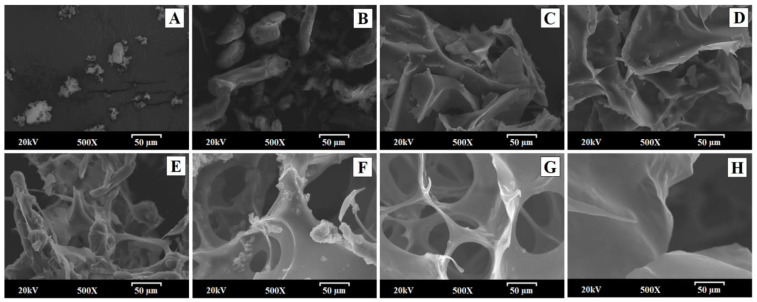
Scanning electron micrographs of the following samples: (**A**) IND—RM; (**B**) PM—1:2.5; (**C**) SD—1:1; (**D**) SD—0:2.5; (**E**) SD—1:2.5; (**F**) hydrogel composed of pure IND (HIND—RM); (**G**) physical mixture hydrogel HPM—1:2.5; and (**H**) solid dispersion hydrogel HSD—1:2.5.

IND—RM presented heterogeneous aggregated crystals of different sizes (Figure 1A) [9]. The smooth fibers and particles of LHPC (Figure 1B) could easily be distinguished from the heterogeneous microcrystalline aggregated, possibly related to IND, in PM—1:2.5 but not in the solid dispersions The original morphology of all the components disappeared in the solid dispersions, while SD-1:1 observed at the same magnification (500×) (Figure 1C) had some small particles covering its surface, possibly attributed to IND in the crystalline form. In contrast, solid dispersions with higher LHPC loadings with and without IND (SD—1:2.5 and SD—0:2.5) resembled a matrix with a scaly surface (Figure 1D and Figure 1E, respectively). It is noteworthy that the solid dispersion without IND (SD—0:2.5) has the same appearance as the formulation with the drug in the same proportion of LHPC (Figure 1D,E). These results showed that from the IND:LHPC ratio of 1:2.5, a flaky structure is present where IND was dispersed homogeneously in the LHPC polymeric chains for solid dispersions, where it could possibly exist in a mostly amorphous form [37].

Scanning electron micrographs of the HIND—RM hydrogel (Figure 1F) revealed a characteristic morphology of crystalline aggregates approximately 5–10 µm in size on a smooth surface. These crystals appeared to be irregular in shape and size, as seen in Figure 1, likely corresponding to IND. The physical mixture of the drug and carrier within the hydrogel in a 1:2.5 ratio showed a highly porous structure with interconnected small pores around 10–30 µm, attributed to the presence of HPG and LHPC within the polymeric network. The presence of the drug in crystalline microaggregate forms, with sizes around 5–10 µm similar to the HIND—RM hydrogel, and some polymeric cellulosic fibers, possibly attributed to LHPC, were also observed in this sample (Figure 1G). In the case of the freeze-dried solid dispersion HSD—1:2.5 hydrogel (Figure 1H), it was difficult to distinguish the presence of IND crystals. IND crystals appeared to be incorporated into the LHPC fibers. The solid dispersion appeared as a highly porous matrix, featuring larger pores approximately 25–50 µm in size. This outcome could be attributed to the dispersion of the drug within the polymeric LHPC chains. Previous studies have shown that the addition of hydrophilic polymers to the hydrogel alters its porous structure [28], resulting in larger pores compared to the other freeze-dried hydrogels. These HPG hydrogels exhibited a highly porous structure, forming channels within the interior of the system. Similar porous structures have been observed in previous studies of freeze-dried topical hydrogels. In other hydrogel formulations, these highly porous structures are formed due to polymer disentanglement in an aqueous medium after the freeze-drying process [38].

### 3.2. Physicochemical Characterization of IND—SD Hydrogels

Differential scanning calorimetry (DSC), X-ray powder diffraction (XRPD), and Fourier-transform infrared spectroscopy (FTIR) were employed for evaluating drug/polymer and polymer/polymer interactions, as well as to investigate changes in crystallinity after the freeze-drying process and to assess the influence of including different ratios of LHPC into the SD hydrogels.

**Figure 2:**
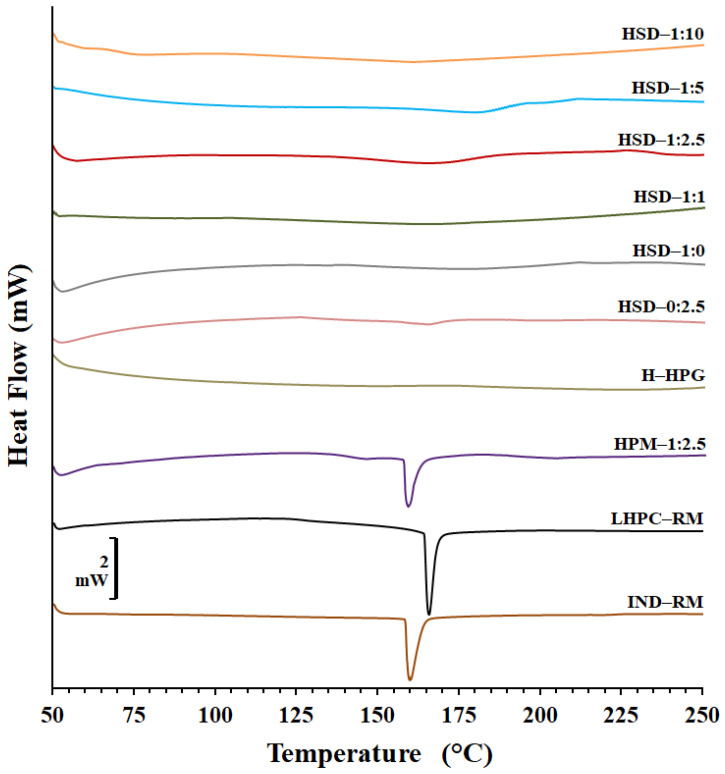
DSC thermograms of IND and LHPC raw materials and physical mixture hydrogel (HPM—1:2.5), HPG hydrogel (H—HPG), and solid dispersion hydrogels (HSD—1:0, HSD—1:1, HSD—1:2.5, HSD—0:2.5, HSD—1:5, and HSD—1:10).

Figure 2 shows the DSC scans for pure IND (IND—RM) and LHPC alone, the physical mixture PM 1:2.5, the physical mixture hydrogel (HPM—1:2.5), and the various solid dispersion hydrogels (HSD—1:0, HSD—1:1, HSD—1:2.5, HSD—1:5, and HSD—1:10).

IND—RM revealed an endothermic peak at 159.90 °C, with an enthalpy of fusion of −116.05 J/g, indicating high crystallinity [39]. Previous studies showed Tg’ values for IND between 50 and 55 °C and a similar endothermic peak (~157 °C) [40]. The thermogram of the LHPC carrier illustrated a melting peak at 166.68 °C (−52.14 J/g), suggesting a semicrystalline nature [31,33].

PM—1:2.5 displayed two broad endothermic peaks: one for IND at 152.9 °C and another at 171.69 °C, ascribed to LHPC. The decrease in enthalpy values and the leftward shift might result from a minor polymorphic transition during the mixing process and a dilution effect of the drug within the carrier [21,41]. Additionally, the temperature difference between the two peaks suggests a strong compatibility between the polymer chains of IND and LHPC [42]. The freeze-dried HPG hydrogel (H—HPG) did not present any endothermic peak due to its lyophilization process, and only one endothermic event at high temperatures (above 300 °C) was observed, which is related to the main chain decomposition and glycosidic linkage cleavage, as reported by other authors [43].

In the case of the freeze-dried hydrogel composed of the physical mixture (HPM—1:2.5), the peak of IND was weakened, broadened, and appeared at 158.21 °C with an enthalpy value of −20.74 J/g, and there was no trace of the LHPC peak. In the freeze-dried solid dispersion hydrogel formulation without IND (HSD—0:2.5), the IND peak disappeared, and there was a slight broad band around 170 °C, replacing the LHPC peak, due to the lyophilization process. Meanwhile, both peaks corresponding to IND and LHPC disappeared completely in the case of hydrogels composed of IND—SDs (HSD—1:0, HSD—1:1, HSD—1:2.5, HSD—1:5, and HSD—1:10). The differences in the thermal behavior of IND hydrogels in the form of PM and SDs suggested that the drug crystallinity decreased when prepared as SDs, probably due to a combination of two factors: the freeze-drying process (major) and the presence of the hydrophilic carrier (minor) [44]. Such an endotherm was not detected in the SD, suggesting the amorphous conversion of IND in the different solid dispersions. Moreover, no other thermal event was detected, which indicates the formation of a single-phase system [45].

XRPD analyses were conducted to investigate changes in crystallinity after the freeze-drying process and to assess the influence of including different ratios of LHPC into the SD hydrogels.

**Figure 3:**
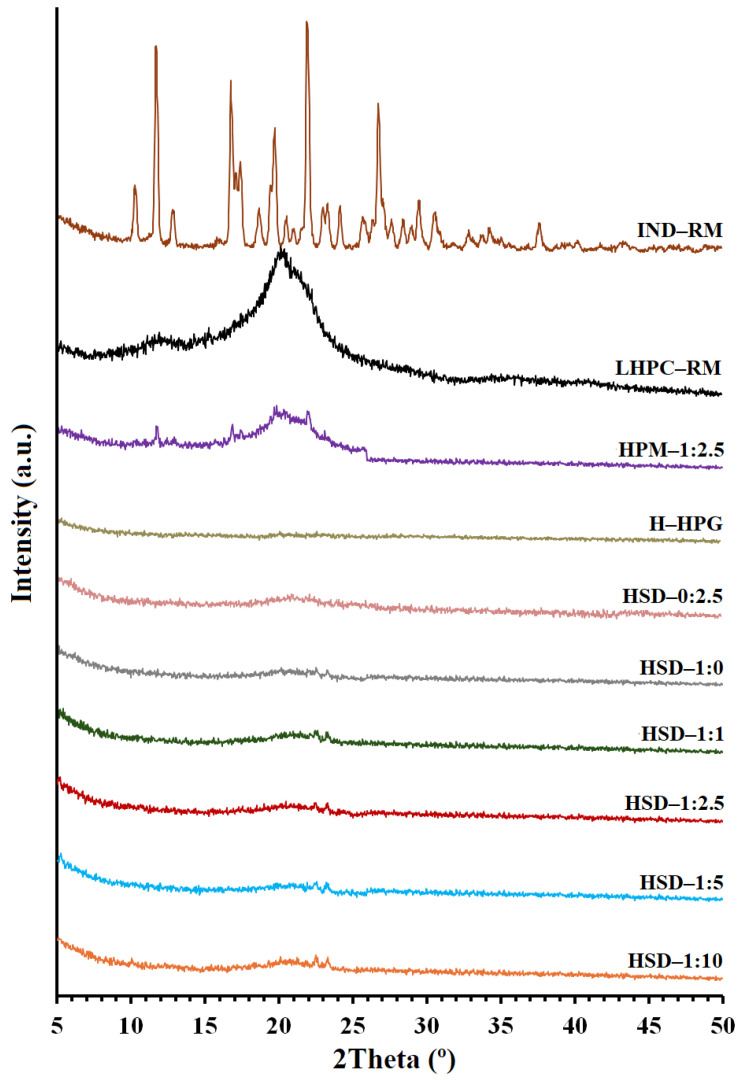
X-ray powder diffraction scans of IND and LHPC raw materials, physical mixture hydrogel (HPM—1:2.5), HPG hydrogel (H—HPG), and solid dispersion hydrogels (HSD—1:0, HSD—1:1, HSD—1:2.5, HSD—0:2.5, HSD—1:5, and HSD—1:10).

The crystal structure of pure IND (Figure 3) showed characteristic sharp peaks at 10.44°, 11.71°, 13.02°, 16.86°, 17.50°, 18.60°, 19.83°, 21.91°, and 26.82° (2θ), corresponding to the γ-crystalline form polymorph of indomethacin [46]. The low intensity of its diffraction peaks suggests that the drug substance has low crystallinity. The XRPD pattern of LHPC showed a unique semicrystalline halo diffraction range between 15.22° and 25.5° (2θ), with the highest diffraction intensity at 20.17° (2θ). [45]. The freeze-dried HPG hydrogel (H—HPG) did not present any semicrystalline halo after its lyophilization process. It did not show the weak semicrystalline halo characteristic at 2θ = 20° reported by other authors in HPG raw non-freeze-dried material [39].

In the case of the hydrogel composed of pure IND (HIND—RM), there was a decrease in the intensity of IND, with only two major peaks remaining at the same positions (11.71° and 21.91°). Meanwhile, the physical mixture hydrogel (HPM—1:2.5) exhibited three distinctive peaks of IND—RM and a broad semicrystalline halo, indicative of the LHPC polymer (Figure 3). The reduction in the intensity and quantity of IND peaks in both formulations, HIND—RM and HPM—1:2.5, could be due to a decrease in drug crystallinity that occurred after the freeze-drying process and also to a dilution effect in the HPM—1:2.5 hydrogel [8]. The use of temperatures lower than the glass transition temperature (Tg’) of IND (Tg’ values around 50–60 °C) observed in previous studies [40,41] favors the vitrification process of IND within the H—HPG network, obtaining samples with low crystallinity. Additionally, a dilution effect in the HPM—1:2.5 hydrogel was observed [8]. In the freeze-dried solid dispersion hydrogel formulation without IND (HSD—0:2.5), the characteristic peaks attributed to IND disappeared, and there was a semicrystalline halo diffraction range between 15.22° and 25.5° (2θ), with lower diffraction intensity, in comparison to LHPC raw material, likely as a result of the lyophilization process.

Nevertheless, in all solid dispersion hydrogels (HSD—1:0, HSD—1:1, HSD—1:2.5, HSD—1:5, and HSD—1:10), the characteristic peaks of IND were absent (see Figure 3), aligning with the DSC study results. Consequently, the drug in the form of SDs exhibited a significant reduction in crystallinity, which could enhance rapid drug dissolution due to the increased Gibbs free energy [8,47]. XRPD and DSC analyses of these solid dispersion hydrogels revealed the presence of the amorphous form of IND, thereby enhancing its solubility at pH 5.8.

**Figure 4:**
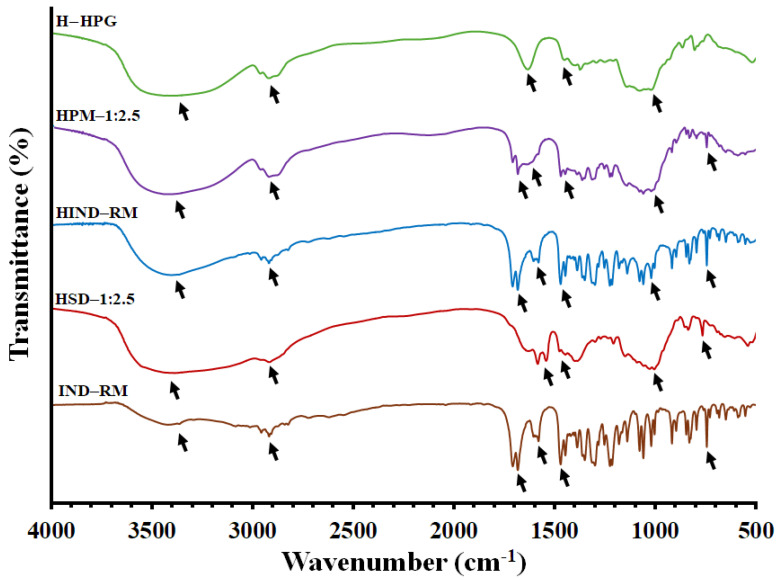
FTIR spectra of the pure IND (IND—RM), HPG hydrogel (H—HPG), hydrogel composed of pure IND (HIND—RM), physical mixture hydrogel HPM—1:2.5, and solid dispersion hydrogel HSD—1:2.5.

FTIR spectroscopy was used for the estimation of the drug/polymer and polymer/water molecular interactions (Figure 4). The FTIR spectrum of IND alone is displayed in Figure 4. IND showed characteristic peaks around 1709 cm^−^^1^ for the presence of (C=O) carboxylic acid, at 3420 cm^−^^1^ for N–H stretching vibrations, at 1583 cm^−^^1^ for (benzoilo)-C=O amide, small and medium stretching at 3088–2833 cm^−^^1^ for C-H (CH2) stretching alkane, small stretching at 854–734 cm^−^^1^ for the (Cl) chlorine group, strong bending at 1477 cm^−^^1^ for C=C stretching, at 914–694 cm^−^^1^ for aromatic stretching, and at 1018–651.89 cm^−^^1^ for (=C-H) alkene stretching (out-of-plane bend) [48].

Based on our previous results, the FTIR spectra of the freeze-dried H—HPG hydrogel indicate the presence of a band at 3236 cm^−1^ related to –OH stretching representative of the HPG polymer/water interaction. Additionally, this formulation showed increased intensity in bands correlated to the –OH stretching vibration from the hydroxyl groups and the C–O–H band, representing hydrogen bonding between the polymer chains and the water molecules [33,49]. Two bands at 1575 and 1389 cm^−1^ were observed, which correspond to broad deformation bands of –OH from aqueous medium. Furthermore, both bands at 2900 and 1027 cm^−1^ were related to the stretching vibrations of C–H and C–O–H, respectively, characteristic of the HPG polymer

Figure 4 shows that the spectra of all tested freeze-dried HPG hydrogels (HIND—RM, HPM—1:2.5, and HSD—1:2.5) are characterized by a wide band (wavenumber at 3740–3000 cm^−1^) corresponding to the stretching vibrations of the hydroxyl group (free OH group from water and hydrogen bonds). The bands around 1575, 1391, and 1029 cm^−1^ were related to –OH and C–O–H deformations caused by the interaction between HPG/water, all of them characteristic of the freeze-dried HPG hydrogel [33,50]. Characteristic bands of IND in both formulations, HIND—RM and HPM—1:2.5, were observed at 1709 cm⁻^1^, 1583 cm⁻^1^, 854–734 cm⁻^1^, 1477 cm⁻^1^, and 914–694 cm⁻^1^, retaining their maximum peaks. These peaks correspond to (C=O) carboxylic acid, (benzoyl)-C=O amide, the (Cl) chlorine group, C=C stretching, and aromatic stretching, respectively. The results indicated that there were no considerable changes in the FTIR peaks of the drug when mixed with the hydrogel, demonstrating the absence of any interaction between IND and the HPG polymer [51].

Lastly, the solid dispersion hydrogel HSD—1:2.5 exhibited a broader band at 3433 cm^−^^1^ compared to other hydrogels, due to the overlap of the N–H band with the –OH band from the HPG hydrogel. Furthermore, the band at 778 cm^−^^1^ corresponding to the chlorine group (Cl) from IND remained unchanged. However, the peaks at 1589, 1410, and 1018 cm^−^^1^, which correspond to the hydroxyl bands and C–O–H stretching vibrations of the freeze-dried HPG hydrogel, were slightly shifted to higher wavenumbers [38]. This shift may result from hydrogen bonding interactions between the –OH groups from both LHPC and HPG polymeric chains and also with water molecules [52].

### 3.3. Viscosity Study of Hydrogels

Measuring the viscosity of hydrogels is essential for determining their application ease, drug release rate, and structural integrity, ensuring the optimal performance of the formulation [53]. It was previously studied that a hydrogel with favorable properties for topical administration was achieved at a concentration of 1.75% (*w*/*v*) [33].

**Figure 5:**
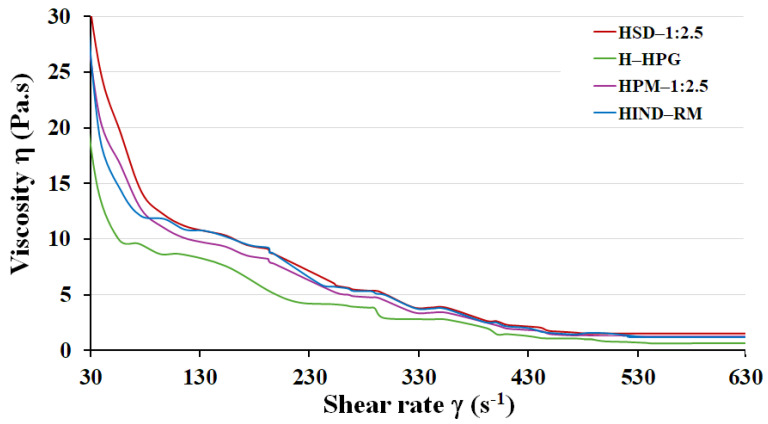
Viscosity behavior of hydrogel formulations: HPG blank hydrogel (H—HPG), hydrogel composed of pure IND (HIND—RM), physical mixture hydrogel HPM—1:2.5, and solid dispersion hydrogel HSD—1:2.5. Results are presented as mean values (n = 3) for each formulation.

Viscosity curves for the prepared HPG-based hydrogels were evaluated, and the structural viscosity at a low shear rate (50 s^−1^) was compared, as illustrated in Figure 5. As can been seen, similar flow profiles were recorded for the tested hydrogels (H—HPG, HIND—RM, HPM—1:2.5, and HSD—1:2.5), the viscosity decreasing with the shear stress increase. This behavior conducts to the pseudoplastic properties of the samples which facilitate the formulations’ flow and consequently their suitable manipulation [54].

Viscosity curves were evaluated, as were four types of hydrogels, one containing IND raw material, the second one containing a physical mixture with IND, the third one containing a solid dispersion with IND, and another one without IND. In all hydrogels, the presence of IND led to an increase in shear stress (Pa), presenting the following viscosity values at 25 °C: 9.96 ± 0.10 Pa·s for H—HPG, 14.37 ± 0.10 Pa·s for HIND—RM, 16.69 ± 0.11 Pa·s for HPM—1:2.5, and 19.52 ± 0.26 Pa·s for HSD—1:2.5. All tested IND-containing hydrogels are principally suitable for topical application. Additionally, various studies have shown that the viscosity of the hydrogels significantly influences the percentage of drug release [55].

Viscosity studies for HPM—1:2.5 demonstrated that adding both IND and LHPC to the HPG hydrogel significantly increased (*p* > 0.05) the viscosity compared to H—HPG. The higher viscosity values may result from the presence of a dispersed phase within the HPG hydrogel [56].

The highest increase in viscosity values (19.52 ± 0.26 Pa·s at 50 s⁻^1^) was observed for HSD—1:2.5 compared to HPM—1:2.5. This increase may be attributed to two factors: firstly, the increased dispersed phase within the SD hydrogel and secondly, to interactions between LHPC and HPG, which likely result in more free hydroxyl groups on the network surface. These groups can facilitate polymer/water interactions, as observed in the FTIR spectra. This enhanced hydrogen bonding could explain the increased viscosity in the solid dispersion hydrogel [28].

### 3.4. Solubility Study of Solid Dispersions

The purpose of this study is to evaluate the influence of different amounts of LHPC on the solubility properties of IND, which could improve its release in the presence of this cellulosic polymer, thus enhancing the topical delivery of IND [57]. In the present study, pH 5.8 was selected for the solubility studies to enable comparison with different studies on the topical administration of hydrogels [14,33].

**Figure 6:**
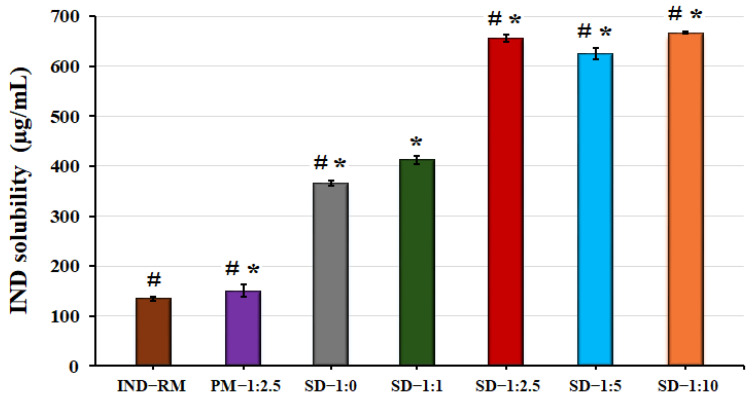
Solubility studies were conducted on IND raw material (IND—RM), solid dispersions at ratios of IND:LHPC 1:0 (SD—1:0), 1:1 (SD—1:1), 1:2.5 (SD—1:2.5), 1:5 (SD—1:5), and 1:10 (SD—1:10), as well as the physical mixture with a IND:LHPC ratio of 1:2.5 (PM—1:2.5) in phosphate buffer (pH 5.8). ANOVA was used for multiple comparison. (*) indicates a significant difference (*p* < 0.05) compared with IND—RM. (#) indicates a significant difference (*p* < 0.05) compared with SD—1:1.

As can be seen in Figure 6, the solubility of IND raw material at this pH (5.8) is 135.08 ± 4.05 µg/mL. The drug solubility coefficient is only slightly improved in the physical mixture (1.12-fold) compared to pure IND. In the DSC studies, PM—1:2.5 showed a crystalline form, and the presence of LHPC in PM—1:2.5 is not sufficient to prevent the initiation of nucleation and precipitation processes around the IND crystals [45].

In all the other SDs, it is increased compared to both IND-RM and PM—IND—1:2.5. The SD—1:0 formulation, without LHPC, has the lowest solubility (365.66 ± 5.34 µg/mL) among the SDs. However, when LHPC is included in the formulation at a low ratio, IND solid dispersion SD—1:1, the solubility of the drug is moderately increased (412.62 ± 8.70 µg/mL) compared to SD—1:0. The solubility results of this IND formulation (SD—1:1), although using sodium dodecyl sulfate (SDS) as a surfactant, showed lower solubility values compared to those obtained in previous studies with meloxicam SD—1:1 [33]. The strong hydrophobicity and poor solubility of IND necessitate the combination of low proportions of SDS as a surfactant with the use of the hydrophilic polymer chains of LHPC to improve the surface wettability of strongly hydrophobic drugs like IND. Starting from a ratio of IND:LHPC 1:2.5, the values of the IND solubility coefficient show a significant increase (*p* < 0.05) by 5-fold compared to those of the IND raw material. At increasing ratios of LHPC, this polymeric carrier did not have any further influence on IND solubility, achieving very similar solubility values in both formulations SD—1:5 and SD—1:10 compared to SD—1:2.5. Probably, the high amount of LHPC polymeric chains forms a more cohesive interpolymeric network, hindering water entrance [19]. These results indicated that in solid dispersions, the lyophilization process favors the formation of an amorphous form of IND, while the use of LHPC maintains a supersaturation concentration above the critical nucleation concentration.

In the solubility studies, the formulations with LHPC experienced an increase in solubility at a determined IND:LHPC ratio (1:2.5). To verify these results, a dissolution rate test was performed in a pH 5.8 medium at 37 °C for all hydrogel formulations.

### 3.5. In Vitro Release Profile Study of Hydrogels

This study is important for evaluating the influence of different IND—SD formulations within the hydrogel matrix on drug release at a pH simulating the skin environment (pH between 5.5 and 6.0) and temperature at 32 ± 1 °C, which could affect its topical delivery.

In a previous study, the gelling agent (HPG) at a concentration of 1.75% (*w*/*v*) was employed, resulting in a hydrogel with good properties for topical administration [33]. For this reason, this HPG concentration, 1.75% (*w*/*v*), was chosen for the different release studies of hydrogel formulations.

**Figure 7:**
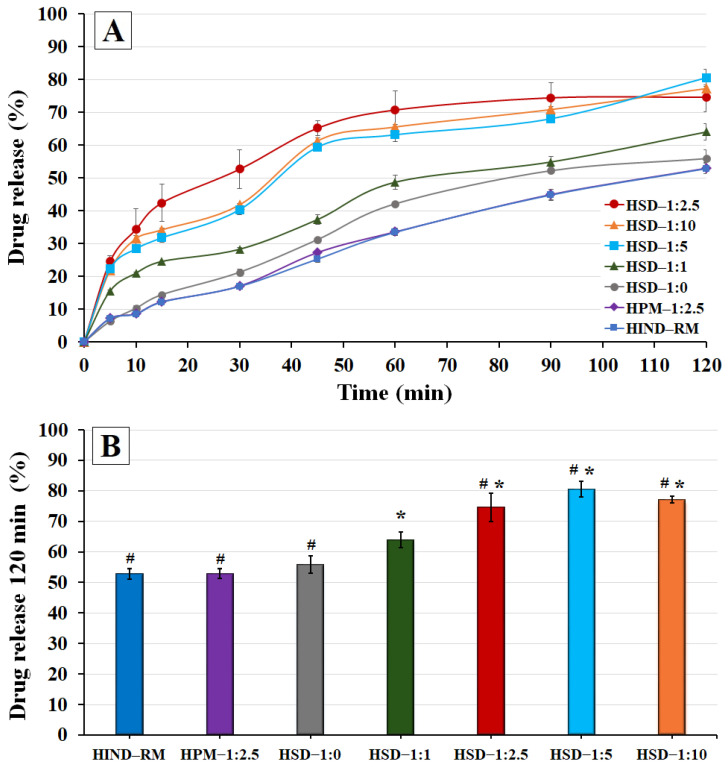
(**A**) Release profiles of IND at pH 5.8 for hydrogels composed of pure IND (HIND—RM), physical mixture hydrogel (HPM—1:2.5), and solid dispersion hydrogels (HSD—1:0, HSD—1:1, HSD—1:2.5, HSD—1:5, and HSD—1:10); (**B**) histogram of drug release (%) at 120 min for different hydrogel formulations (mean ± standard deviation, n = 4). ANOVA was used for multiple comparison. (*) indicates significant difference (*p* < 0.05) compared with HIND—RM, and (#) indicates significant difference (*p* < 0.05) compared with HSD—1:0.

The release rate profiles of both hydrogels HIND—RM and HPM—1:2.5 were very analogous, both of them showing a gradual and slow release rate up to 2 h, achieving similar values (*p* > 0.05) of 53.17 ± 1.65% and 52.80 ± 1.58%, respectively (Figure 7A,B). Slow release profiles for poorly soluble drugs like IND have been similarly reported in other studies [58]. These results are consistent with the solubility studies, where the physical mixture exhibited a solubility value similar to that of pure IND. Nonetheless, the presence of hydrophilic swellable additives such as LHPC (HPM—1:2.5) did not show a significant improvement (*p* > 0.05) compared to IND-RM at 120 min. However, in previous studies of meloxicam hydrogels, the presence of a hydrophilic polymer such as LHPC in the physical mixture improved surface wettability and significantly increased (*p* < 0.05) the meloxicam release rate from hydrogels during the initial times [33]. This lower release profile in HPM—1:2.5 and its low release percentage at 2 h (52.80 ± 1.58%) observed in Figure 7B are related to the strong hydrophobic nature of IND, which requires the use of lyophilized solid dispersions with an association of SDS as a wetting agent, and high ratios of LHPC as a hydrophilic carrier, to significantly increase the IND release rate from hydrogels. In previous DSC studies, these samples exhibited crystalline forms for IND that were related to these slow release profiles. [21]. The solid dispersion hydrogel HSD—1:0 exhibited a modest enhancement in its release profile compared to both HIND—RM and HPM—1:2.5, achieving a 1.26-fold increase in release percentages at 1 h. This slight improvement can likely be attributed to the inclusion of SDS in HSD—1:0 and the freeze-drying process, which together may improve the surface wettability and moistening of the drug, thereby increasing its release [59]. However, at 2 h (see Figure 7B), the release percentages of HSD—1:0 were not significant (*p* > 0.05) compared to HIND—RM and HPM—1:2.5. Nevertheless, this formulation without LHPC (HSD—1:0) resulted in a slightly lower release profile compared to other solid dispersion hydrogels. Previous studies have indicated that including hydrophilic carriers in SDs can notably improve the solubility and wettability of hydrophobic drugs with low solubility [60].

The hydrogels containing solid dispersions with low ratios of LHPC, HSD-1:1 (Figure 7A), exhibited a significant increase (*p* < 0.05) of 1.67- and 1.46-fold at 30 min and 1 h, respectively, compared to HIND—RM. Additionally, a significant improvement (*p* < 0.05) in the release percentages at 2 h compared to SD-1:0 was observed (see Figure 7B). The freeze-dried process with hydrophilic polymers such as LHPC was carried out at freezing temperatures below the glass transition temperature (Tg’), favoring the vitrification of the amorphous form of IND, which favors the rapid release of 1:1 SD. A similar vitrification of amorphous forms below the freezing glass transition temperature (Tg’) has been observed in studies with other hydrophilic polymers and IND [41]. These results indicate that the LHPC chains within the network produce drug/polymer interactions by hydrogen bonding, allowing for increased interface wetting and an improved release rate [60].

The solid dispersion hydrogels HSD—1:2.5, HSD—1:5, and HSD—1:10 demonstrated a remarkable increase (*p* < 0.05) in the release studies compared to HIND—RM. Moreover, drug release in these solid dispersions showed similar release profiles. Thus, SD—1:2.5 showed increases at 30 min (3.11-fold) and at 1 h (2.11-fold), compared to the HIND—RM hydrogel (Figure 7A). This result was confirmed in Figure 7B where significant differences (*p* < 0.05) were observed between the solid dispersions with high ratios of LHPC (HSD—1:2.5, HSD—1:5, and HSD—1:10), and the hydrogels containing solid dispersions with low ratios of LHPC (HSD—1:1). Probably, the inclusion of high amounts of hydrophilic LHPC chains within the network enhanced the wettability of the hydrophobic IND particles’ surface, thus improving their solubility and release profile [61]. Finally, in the dissolution studies at 2 h (see Figure 7B), the dissolution results for both HSD—1:5 and HSD—1:10 did not show significant improvements (*p* > 0.05) compared to HSD—1:2.5. In all these solid dispersions, the high number of LHPC chains within the interpolymer network produces a significant separation of the hydrophilic chains and increases the wettability capacity on the surface of the IND particles to enter the solution from the dissolution medium [60].

Moreover, it is observed that with IND:LHPC ratios greater than 1:2.5, in the solid dispersion hydrogels HSD—1:5 and HSD—1:10, there is a delay in the dissolution of IND during the initial times, between 15 and 30 min. This phenomenon is likely attributed to the higher concentration of the hydrophilic carrier, which prevents water from entering the formulation, thus delaying the solubilization of the drug. Additionally, the dissolution results for both HSD—1:5 and HSD—1:10 did not show significant improvements compared to HSD—1:2.5 at 120 min. This result observed during the initial dissolution times is likely due to the higher number of LHPC chains within the interpolymer network, which prevented further enhancement in the IND release rate [46].

### 3.6. Cytotoxicity Analysis of Hydrogel Formulations

The importance of conducting cytotoxicity studies lies in ensuring the safety and biocompatibility of new drug formulations [62]. After 24 h of exposure, none of the formulations tested, neither the IND-RM nor vehicles, were cytotoxic on the different cells’ lines. Table 1 shows the percentage of cytotoxicity (%C) at the highest concentration evaluated (200 µM) against the four mammalian cell lines (Vero CCL-81, macrophages J774, HeLa cells, and L929 fibroblasts). The cytotoxic concentration CC50 was not calculated due to the absence of cytotoxicity.

## 4. Conclusions

Hydroxypropyl guar (HPG) hydrogels at a concentration of 1.75% (*w*/*v*) with solid dispersions of various IND:LHPC ratios, 1:1; 1:2.5; 1:5; and 1:10, were developed and characterized. This study demonstrated that dispersions of IND into a hydrophilic carrier like LHPC changed the crystallinity of IND. The formation of the IND—LHPC solid dispersion almost completely destroyed the crystallinity of the drug, as shown by the DSC and XRD studies, and represents a suitable modification for improving its topical delivery. The drug solubility coefficient for SD—1:2.5 showed a significant increase (*p* < 0.05) by 5-fold compared to that of the IND raw material. At increasing amounts of LHPC, this polymeric carrier did not have any further influence on IND solubility. The in vitro release of the drug from the IND hydrogel (HIND—RM) demonstrated a gradual release pattern, typical of drugs with low solubility. Possibly, in the case of the HSD—1:2.5 hydrogel, several factors such as the presence of hydrogen bonding in the solid dispersion, the inclusion of hydrophilic carriers like LHPC, and the amorphization of the drug contributed to the enhanced dissolution of IND, as observed in DSC, XRD, viscosity, SEM, and FTIR studies. Finally, no cytotoxic effects were observed against several types of mammalian cells, indicating that the solid dispersion hydrogel HSD—1:2.5 is biocompatible and non-toxic. These results suggest that SD-IND hydrogel formulations could be promising candidates for the topical delivery of IND in HPG hydrogels.

## Figures and Tables

**Table 1:** Unspecific cytotoxicity for IND raw material (IND—RM) and hydrogels composed of pure IND (HIND—RM), physical mixture (HPM—1:2.5), solid dispersion (HSD—1:2.5), and blank HPG hydrogel (H—HPG) in four different mammalian cells.

Formulations	Unspecific cytotoxicity			
	Vero	Macrophages	L929	HeLa
IND—RM	0	8.04 ± 0.26	11.87 ± 1.45	4.81 ± 0.26
HIND—RM	0	7.90 ± 1.44	10.85 ± 0.34	25.97 ± 0.83
HPM—1:2.5	0	5.07 ± 0.86	15.18 ± 1.82	7.46 ± 2.29
HSD—1:2.5	6.74 ± 1.13	8.62 ± 1.09	10.95 ± 0.50	9.46 ± 1.47
H—HPG	0	0	10.38 ± 2.24	0

## Data Availability

The data presented in this study are openly available in this article.
